# Proteomic mechanistic profile of patients with diabetes at risk of developing heart failure: insights from the HOMAGE trial

**DOI:** 10.1186/s12933-021-01357-9

**Published:** 2021-08-09

**Authors:** Job A. J. Verdonschot, João Pedro Ferreira, Pierpaolo Pellicori, Hans-Peter Brunner-La Rocca, Andrew L. Clark, Franco Cosmi, Joe Cuthbert, Nicolas Girerd, Beatrice Mariottoni, Johannes Petutschnigg, Patrick Rossignol, John G. F. Cleland, Faiez Zannad, Stephane R. B. Heymans, HOMAGE “Heart Omics in AGEing” consortium

**Affiliations:** 1 Department of Clinical Genetics, Maastricht University Medical Center, Maastricht, The Netherlands; 2 Centre d’Investigations Cliniques Plurithématique 1433, Université de Lorraine, Institut National de la Santé et de la Recherche Médicale 1116, Centre Hospitalier Régional Universitaire de Nancy, French Clinical Research Infrastructure Network, Investigation Network Initiative-Cardiovascular and Renal Clinical Trialists, Nancy, France; 3 Robertson Centre for Biostatistics, Institute of Health and Wellbeing, University of Glasgow, Glasgow Royal Infirmary, Glasgow, G12 8QQ UK; 4 Department of Cardiology, Maastricht University Medical Center (MUMC+), PO Box 5800, 6202 AZ Maastricht, The Netherlands; 5 Department of Cardiology, University of Hull, Castle Hill Hospital, Cottingham, East Riding of Yorkshire, UK; 6 Department of Cardiology, Cortona Hospital, Arezzo, Italy; 7 Department of Internal Medicine and Cardiology, Campus Virchow Klinikum, Charite´ University Medicine Berlin, Berlin Institute of Health (BIH), and German Centre for Cardiovascular research (DZHK), Partner Site Berlin, Berlin, Germany

**Keywords:** Diabetes, Biomarker, Heart failure, Spironolactone, Fibrosis

## Abstract

**Background:**

Patients with diabetes mellitus (DM) are at increased risk of developing heart failure (HF). The “Heart OMics in AGEing” (HOMAGE) trial suggested that spironolactone had beneficial effect on fibrosis and cardiac remodelling in an at risk population, potentially slowing the progression towards HF. We compared the proteomic profile of patients with and without diabetes among patients at risk for HF in the HOMAGE trial.

**Methods:**

Protein biomarkers (n = 276) from the Olink®Proseek-Multiplex cardiovascular and inflammation panels were measured in plasma collected at baseline and 9 months (or last visit) from HOMAGE trial participants including 217 patients with, and 310 without, diabetes.

**Results:**

Twenty-one biomarkers were increased and five decreased in patients with diabetes compared to non-diabetics at baseline. The markers clustered mainly within inflammatory and proteolytic pathways, with granulin as the key-hub, as revealed by knowledge-induced network and subsequent gene enrichment analysis. Treatment with spironolactone in diabetic patients did not lead to large changes in biomarkers. The effects of spironolactone on NTproBNP, fibrosis biomarkers and echocardiographic measures of diastolic function were similar in patients with and without diabetes (all interaction analyses p > 0.05).

**Conclusions:**

Amongst patients at risk for HF, those with diabetes have higher plasma concentrations of proteins involved in inflammation and proteolysis. Diabetes does not influence the effects of spironolactone on the proteomic profile, and spironolactone produced anti-fibrotic, anti-remodelling, blood pressure and natriuretic peptide lowering effects regardless of diabetes status.

*Trial registration* NCT02556450.

**Supplementary Information:**

The online version contains supplementary material available at 10.1186/s12933-021-01357-9.

## Background

Patients with diabetes mellitus (DM) have an increased risk of developing heart failure (HF) [1] but the mechanisms are poorly established. A better understanding of the pathophysiological processes linking DM and HF may help guiding treatment strategies and potentially reveal novel therapeutic targets [2–4].

The HOMAGE (Heart OMics in AGEing) trial showed that treatment with spironolactone (vs. usual care) in patients at risk of developing HF led to a decrease in collagen synthesis markers, N-terminal pro brain natriuretic peptide (NT-proBNP), reduced blood pressure and improved cardiac remodelling [5, 6]. Small studies have suggested that spironolactone could impair endothelial function and increase glycated hemoglobin (HbA1c) in patients with DM [7, 8]. As spironolactone is a steroidal mineralocorticoid receptor antagonist (MRA) that is not specific for the mineralocorticoid receptor, it may exert other effects (e.g., cortisol elevation) on glucose and insulin metabolism. In a previous analysis we showed the influence of spironolactone on the proteome in the total HOMAGE trial population [9]. It remains unknown to what extent DM influences the effects of spironolactone on clinical and proteomic variables.

In this pre-specified secondary analysis of the HOMAGE trial, we aimed to (1) compare the clinical characteristics of patients with and without DM; (2) study the biomarker profiles, networks and pathways associated with DM at baseline, as compared to patients without diabetes; (3) to assess the effect of spironolactone on the circulating proteomic biomarkers in patients with DM; and (4) to explore whether the DM status might have modified the effect of spironolactone on the main outcomes assessed in the HOMAGE trial (level of collagen synthesis markers, NT-proBNP, echocardiographic measures of cardiac structure and function, and signs/symptoms).

## Methods

### Trial design and population

The HOMAGE trial had a prospective, randomised, open-label, blinded-endpoint (PROBE), multicentre design. People at increased risk of developing HF were randomly assigned to receive either spironolactone or standard care (ClinicalTrials.gov Identifier: NCT02556450). The rationale, trial design and main results have been published [5, 6]. The study was approved by all relevant ethics committees and regulatory bodies. All participants provided written informed consent prior to study-specific procedures. The main participating criteria included age 65 or older (amended to 60 years during the course of the trial), cardiovascular risk defined by the presence of coronary artery disease OR at least 2 of the following: diabetes, treated hypertension, microalbuminuria, abnormal ECG, and a NT-pro BNP between 125 and 1000 ng/l or BNP between 35 and 280 ng/l. The main exclusion criteria were glomerular filtration rate (eGFR) < 30 ml/min/1.73 m^2^, serum potassium > 5.0 mmol/l, left ventricular ejection fraction < 45%, a diagnosis of HF or treatment with loop diuretics, and atrial fibrillation/flutter. For this secondary analysis, patients were divided into two groups based on the presence, or absence, of a diagnosis of DM at baseline.

### Proteomic biomarkers

Baseline and month 9 (or last visit) plasma samples were analysed for 276 protein biomarkers by the TATAA-biocenter using the Olink Proseek® Multiplex cardiovascular (CVD) II, CVD III, and inflammation panels. The proteins were determined using high-throughput Olink Proseek® Multiplex 96 × 96 kits, which measures 92 manually-selected proteins simultaneously in 1 µl of plasma per kit. Each kit uses a proximity extension assay (PEA) technology with dual-recognition DNA-coupled readout, where 92 oligonucleotide-labelled antibody probe pairs are allowed to bind to their respective targets in the sample. The platform provides Log2 normalized protein expression (NPX) values with relative quantification. A detailed description of the Olink® technology is depicted on the website: https://www.olink.com/. The abbreviations, full names and respective Olink® multiplex panels of the studied proteins are described in Additional file 1: Table S1. The assays were performed in a blinded fashion to the treatment allocation. The proteomic results were then merged into the database.

### Statistical analyses

We compared the characteristics of the patients with and without diabetes using the appropriate tests for continuous and categorical variables. To assess whether the biomarkers were expressed differently in patients with and without diabetes, we performed logistic regression analyses with diabetes as outcome variable and adjusting for age, sex, coronary artery disease, hypertension, body mass index and estimated glomerular filtration rate. Since proteins were measured using NPX values on a log2 scale, the odds ratio for each protein estimates the increase in the odds of diabetic status associated with a doubling in the protein concentration. We corrected the findings for multiple testing using a false discovery rate of 5%. After selecting the proteins with differential expression by diabetes status, we tested whether spironolactone could change the levels of the proteins. For consistency with the primary report, we used analysis of covariance (ANCOVA) comparing the difference in any changes between the control and spironolactone groups in the regression model [6].

A linear regression model was fitted, with the protein change (from baseline to last visit) as outcome variable, a binary variable to indicate the treatment group (control/spironolactone), and the baseline protein value (NPX) as covariates. The treatment effect was the coefficient that resulted from the comparison of spironolactone vs. control in the regression model. Residual analysis was used to examine the fit of the model. No data transformation was required to meet the assumptions of linear regression. Similar analyses were performed for the protein change at 1 month. To study if the diabetes status could influence the response to spironolactone on the main outcomes of the study, we performed an ANCOVA analysis with a treatment-by-diabetes interaction term. Statistical analyses were performed using Stata® (version 16, StataCorp LP).

### Bioinformatical and network analyses

We used knowledge-based network analysis with induced network approach by consensuspathDB (CPDB) online server (accessed on 24 November 2020) from Max Planck Institute for Molecular Genetics to identify the links among all significantly different protein biomarkers, based on knowledge of interactions (protein interactions, genetic interactions and biochemical interactions) [7]. The network analysis also identifies additional proteins limited to the first-degree interactors (intermediate nodes) linking our input proteins (seed nodes), with exclusion of low-confidence interactions and quantified by a z-score ≤ 20 calculated for each intermediate node. The Search Tool for the Retrieval of Interacting Genes/Proteins (STRING) database was used to analyse functional enrichment (GO biological processes and KEGG pathways) using proteins that were significantly increased or decreased in diabetic patients compared to non-diabetics at baseline.

## Results

### Clinical characteristics of the study population

Among the 527 patients included in the HOMAGE trial, 217 (41%) had diabetes. Compared to those without diabetes, patients with diabetes had a higher BMI, a higher blood pressure and heart rate (Table 1), were more likely to have a history of hypertension (93% vs. 68%; p < 0.001), but less likely to have a history of coronary artery disease (47% vs. 90%; p < 0.001). Among patients with diabetes, 176 (81%) were on metformin, and 38 (18%) were insulin-dependent. Left ventricular mass was greater (99 [84–114] vs. 91 [79–110] g/m^2^; p = 0.005) and E/e′ ratio higher (10.1 [8–12] vs. 8.9 [7–11]; p < 0.001) in patients with diabetes; however, left atrial volume and NT-proBNP were similar.

**Table 1:** Baseline characteristics according to diabetic status

Variable	Level	Patients without diabetes (n = 310)	Patients with diabetes (n = 217)	p-value
Age (years)		71.9 [68.5–78.1]	73.5 [69.0–79.0]	0.064
Male gender		235 (75.8%)	157 (72.4%)	0.37
Smoking status	Never	102 (32.9%)	76 (35.0%)	0.90
	Ex	177 (57.1%)	123 (56.7%)	
	Current	28 (9.0%)	16 (7.4%)	
	Missing	3 (1.0%)	2 (0.9%)	
Body mass index (kg/m^2^)		27.3 [25.0–29.9]	29.7 [26.5–33.9]	< 0.001
Systolic blood pressure (mmHg)		139.0 [124.0–154.0]	141.0 (132.0–157.0]	0.013
Diastolic blood pressure (mmHg)		79.0 [72.0–86.0]	77.0 (71.0–84.0]	0.097
Heart rate (pm)		59.0 [54.0–65.0]	64.0 (57.0–70.0]	< 0.001
Number of shuttles completed (shuttle-walk test)		54 [35–71]	44 [26–61]	< 0.001
Comorbidities				
Coronary artery disease		278 (89.7%)	101 (46.5%)	< 0.001
Myocardial infarction		163 (58.6%)	51 (50.0%)	0.13
PCI		196 (70.5%)	69 (67.6%)	0.59
CABG		97 (34.9%)	39 (38.2%)	0.55
Hypertension		211 (68.1%)	202 (93.1%)	< 0.001
Stroke/TIA		14 (4.5%)	14 (6.5%)	0.33
COPD		22 (7.1%)	11 (5.1%)	0.34
Medication				
Antiplatelet therapy		267 (86.1%)	147 (67.7%)	< 0.001
Beta blocker		239 (77.1%)	127 (58.5%)	< 0.001
ACE-inhibitor		157 (50.6%)	118 (54.4%)	0.40
Angiotensin receptor blocker		77 (24.8%)	68 (31.3%)	0.10
Calcium channel blocker		56 (18.1%)	54 (24.9%)	0.058
Thiazides		33 (10.6%)	54 (24.9%)	< 0.001
Statin/lipid lowering drug		269 (86.8%)	166 (76.5%)	0.002
Insulin		0 (0.0%)	38 (17.5%)	< 0.001
Metformin		0 (0.0%)	176 (81.1%)	< 0.001
Sulfunylureas		0 (0.0%)	41 (18.9%)	< 0.001
DPP4-inhibitor		0 (0.0%)	33 (15.2%)	< 0.001
Echocardiography				
Left ventricular ejection fraction (%)		62.8 [56.5–66.0]	63.1 [59.2–66.9]	0.35
Left ventricular mass (g/m^2^)		91.0 [78.5–109.9]	99.0 [84.2–114.1]	0.005
Left ventricular hypertrophy		106 (34.2%)	87 (40.1%)	0.17
Left atrial volume (g/m^2^)		30.8 [26.0–36.5]	29.4 [25.1–35.6]	0.32
E/e′ ratio		8.9 [6.9–11.1]	10.1 [8.3–12.0]	< 0.001
E/A ratio		0.8 [0.7–1.0]	0.8 [0.7–1.0]	0.21
TAPSE (mm)		21.4 [16.7–26.3]	22.5 [17.5–26.8]	0.28
MAPSE (mm)		15.5 [13.6–17.7]	15.0 [13.1–17.4]	0.38
Laboratory				
eGFR (ml/min/1.73 m^2^)		72.0 [62.0–82.9]	72.8 [59.0–86.5]	0.75
Urea (mmol/l)		7.5 [5.6–13.2]	10.7 [6.4–15.7]	< 0.001
Haemoglobin (g/dl)		14.2 [13.3–15.0]	13.8 [12.8–14.7]	0.002
Sodium (mmol/l)		140.0 [138.0–141.0]	139.0 [137.0–141.0]	0.052
Potassium (mmol/l)		4.3 [4.1–4.6]	4.3 [4.0–4.5]	0.12
NT-proBNP, ng/l		205 [129–350]	229 [146–373]	0.25
Galectin-3, ng/ml		15.4 [13.1–18.9]	17.4 [14.4–20.9]	< 0.001
High-sensitivity Troponin T, pg/ml		11.6 [8.2–16.0]	14.1 [10.3–21.1]	< 0.001

Values are median [interquartile range] or n (%)

*PCI *percutaneous coronary intervention, *CABG *coronary artery bypass grafting, *TIA *transient ischemic attack, *COPD *chronic obstructive pulmonary disease, *ACE *angiontensin-converting enzyme, *DPP4 *dipeptidyl peptidase-4, *TAPSE *triscupid annular plane systolic excursion, *MAPSE *mitral annular plane systolic excursion, *eGFR *estimated glomerular filtration rate

### Proteomic profile at baseline

Compared to patients without diabetes, those with diabetes had greater expression of 21 proteins and lower expression of 5 proteins after adjustment for age, sex, hypertension, body mass index, coronary artery disease, estimated glomerular filtration rate and multiple test correction at a FDRq < 0.05 level (Table 2).

**Table 2:** Odds ratio of the proteins which are significantly associated with diabetes after adjustment for age, sex, hypertension, BMI, CAD and eGFR

Protein	Abbreviation	Odds ratio (95% CI)	p-value	FDRq
Positive association (n = 21)				
Interleukin-1 receptor type 1	il1rt1	4.52 (2.41–8.5)	0.00001	0.0005
Cathepsin d	ctsd	3.49 (2.13–5.72)	0.00001	0.0005
Growth differentiation factor 15	gdf15	3.41 (2.39–4.87)	0.00001	0.0005
Galectin-4	gal4	2.95 (1.95–4.45)	0.00001	0.0005
E-selectin	sele	2.38 (1.64–3.45)	0.00001	0.0005
Eotaxin	ccl11	2.96 (1.79–4.9)	0.00002	0.0009
Leukemia inhibitory factor receptor	lifr	4.4 (2.15–9.04)	0.00005	0.0017
Kidney injury molecule	kim1	1.79 (1.35–2.38)	0.00005	0.0017
Cathepsin z	ctsz	3.1 (1.78–5.41)	0.00007	0.0019
Hydroxyacid oxidase 1	haox1	1.44 (1.2–1.72)	0.00007	0.0019
P-selectin glycoprotein ligand 1	psgl1	4.23 (1.95–9.16)	0.00026	0.0054
Interleukin-18 receptor 1	il18r1	2.35 (1.43–3.87)	0.00074	0.014
Prostasin	prss8	2.97 (1.55–5.69)	0.00102	0.0173
C–C motif chemokine 15	ccl15	2.13 (1.34–3.38)	0.00135	0.0215
Interleukin-10	il10	1.74 (1.23–2.45)	0.00168	0.024
Gastric intrinsic factor	gif	1.34 (1.11–1.6)	0.00196	0.0253
Matrix metalloprotease 7	mmp7	1.92 (1.27–2.9)	0.00187	0.0253
Granulin	grn	2.66 (1.42–5)	0.00237	0.0292
Follistatin	fs	2.16 (1.29–3.61)	0.00333	0.0392
Alpha-l-iduronidase	idua	2.04 (1.26–3.29)	0.0036	0.0407
Osteoprotegerin	opg	2.43 (1.32–4.45)	0.00422	0.044
Negative association (n = 5)				
Collagen type 1 alpha 1	col1a1	0.33 (0.19–0.58)	0.00013	0.003
Procollagen type 1 carboxy-terminal propeptide	picp	0.36 (0.22–0.62)	0.00016	0.0036
Lipoprotein lipase	lpl	0.48 (0.31–0.74)	0.00085	0.0154
Matrix extracellular phosphoglycoprotein	mepe	0.43 (0.26–0.73)	0.00167	0.024
C-X-C motif chemokine 10	cxcl10	0.65 (0.48–0.87)	0.00418	0.044

**Fig. 1:**
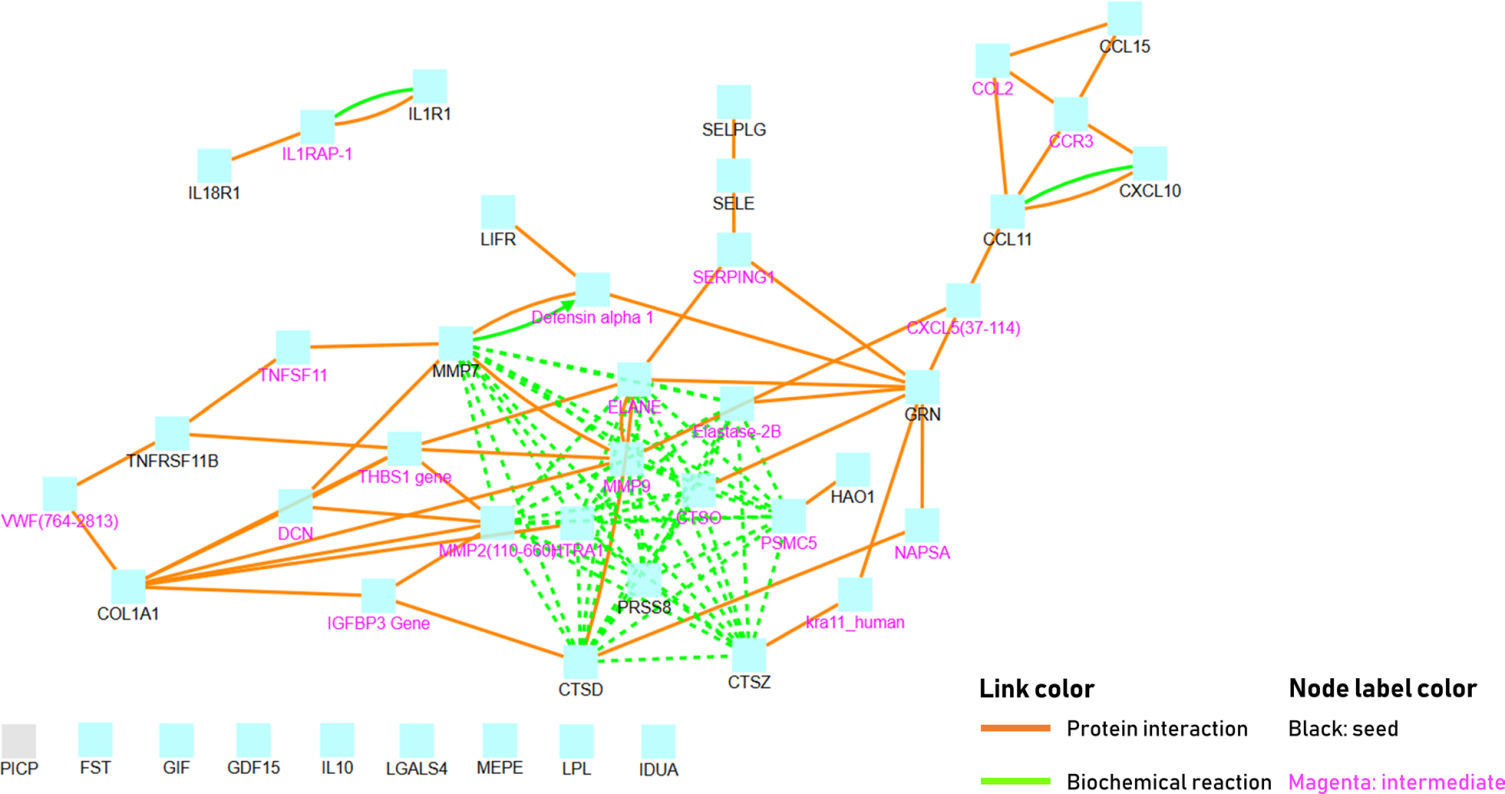
Network of protein biomarkers which were significant differentially detected in patients with diabetes compared to non-diabetics. Knowledge-based induced network with the plasma protein biomarkers which were significantly increased or decreased in diabetic patients (FDRq < 0.05) and their intermediates. The full names of biomarkers are depicted in Additional file 1: Table S1. Additionally, 1 collagen metabolism plasma biomarker (PICP) is also included in this network

**Fig. 2:**
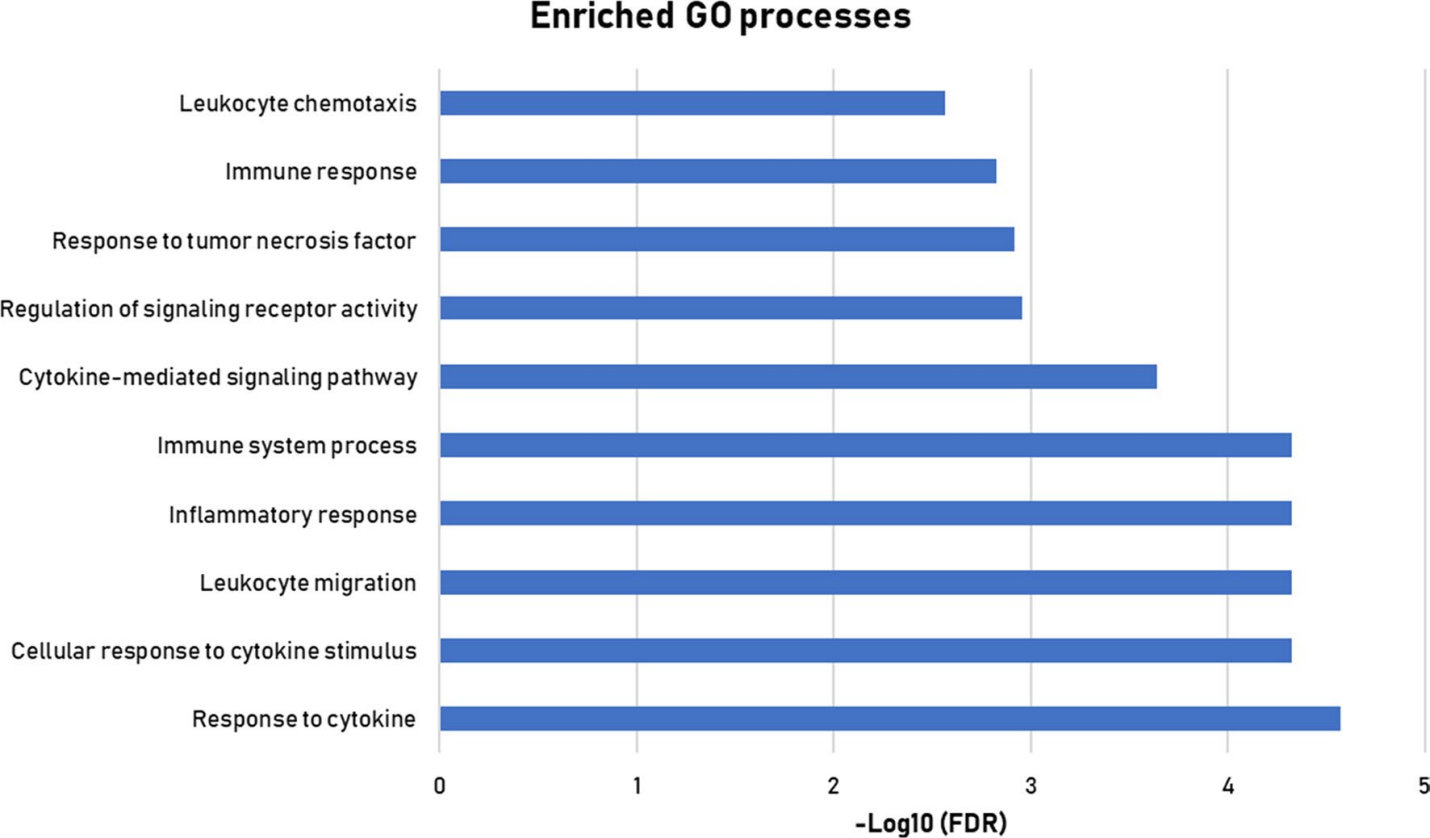
Overrepresented pathways of protein biomarkers in diabetic patients when compared to non-diabetic patients. The gene ontology (GO) biological processes are used as reference. Additional file 1: Table S2 contains further details on the proteins in these pathways

The results of the network analysis are shown in Fig. 1. The central core of the network is characterized by (lysosomal) protease activity reflected in matrix metalloproteinase 7 (MMP7), cathepsin D (CTSD), cathepsin Z (CTSZ) and prostasin (PRSS8). A large number of edges and nodes connect through granulin (GRN), placing this protein as an important hub connecting the proteases with the chemokines [cluster of chemokine (CC-motif) ligand (CCL) 11 and 15 and chemokine (CXC motif) ligand (CXCL) 10]. Pathway enrichment analysis using the Kyoto Encyclopedia of Genes and Genomes (KEGG) terms placed 13 proteins in a biological pathway involving inflammation or lysosomal proteolytic activity (Table 3). An additional analysis using the Gene Ontology terms, showed that the top 10 enriched pathways were all implicated in inflammation (Fig. 2; Additional file 1: Table S2).

**Table 3:** Pathway enrichment analysis using KEGG terms

KEGG-term	Pathway	FDRq	Included proteins
hsa04060	Cytokine–cytokine receptor interaction	0.0000014	*CCL23*, *CCL15*, *CCL11*, *CXCL10*,* LIFR*, *IL10*, *IL1R1*, *OPG*, *IL18R1*
hsa04668	TNF signaling pathway	0.031	*CXCL10*, *SELE*, *IL18R1*
hsa04142	Lysosome	0.039	*CTSD*, *CTSZ*, *IDUA*
hsa04062	Chemokine signaling pathway	0.084	*CCL11*, *CCL15*, *CXCL10*

### Proteomic changes after spironolactone in diabetes patients

Three proteins fell significantly (COL1A1, PICP and SELE) and one protein increased (MMP7) after 9 months of spironolactone treatment (Additional file 1: Table S3). Overall, there was no prominent effect of spironolactone on the proteomic markers in diabetic patients. The activity of the biological processes at baseline in diabetic patients (Table 3; Fig. 2) was unaltered after 9 months of spironolactone treatment.

### Interaction of diabetes status with the main trial outcomes

The primary and secondary outcomes measures in the HOMAGE trial were: PICP and PIIINP (markers of collagen synthesis), NT-proBNP, systolic and diastolic blood pressure, echocardiographic left atrial volume and left ventricular mass. The presence of diabetes did not modify the effect of spironolactone on any of those outcomes (Additional file 1: Table S4), indicating that there was no superior or inferior effect of spironolactone on serum markers of collagen metabolism, cardiac structure and function in patients with DM.

## Discussion

**Fig. 3:**
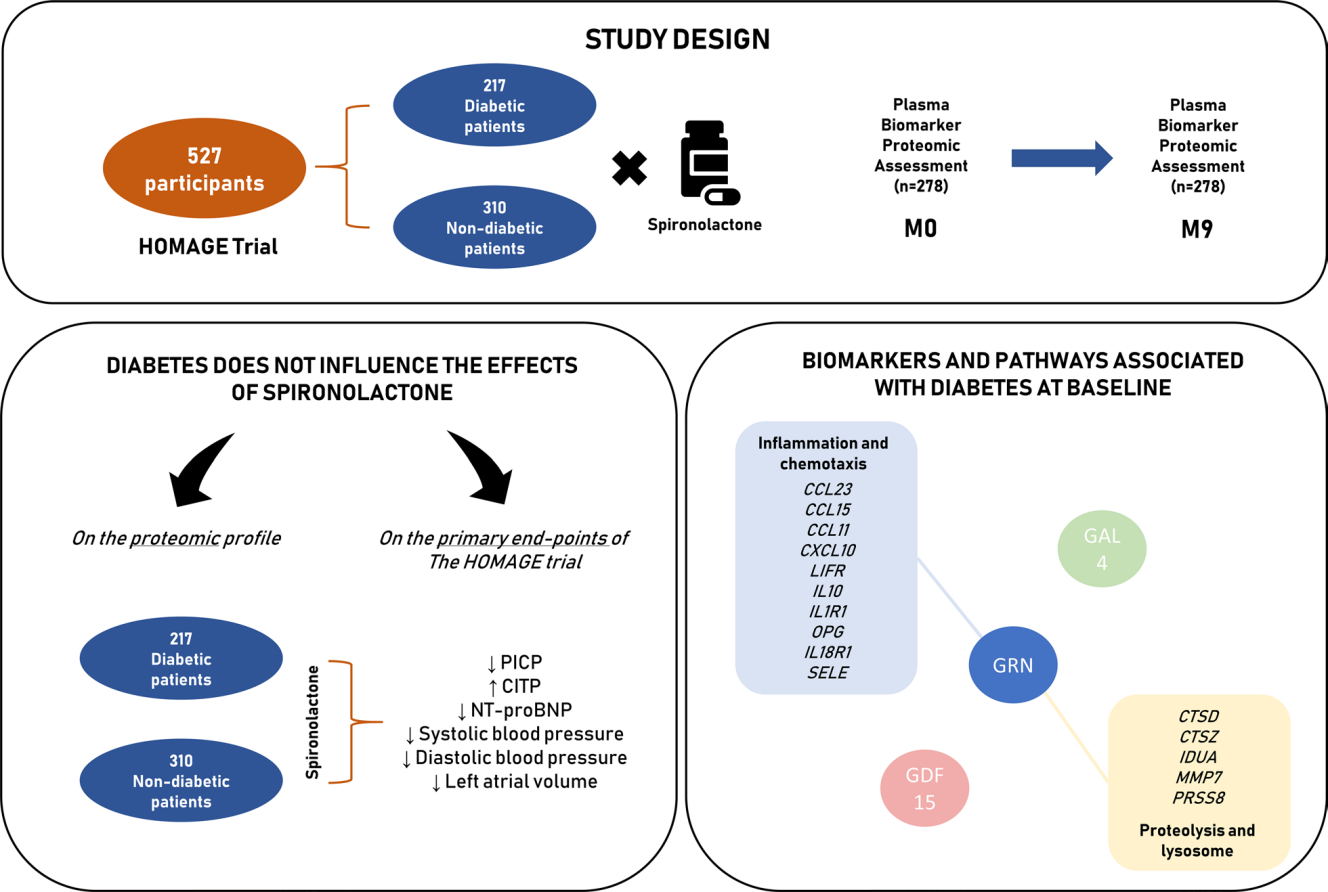
Summary of the study design and results

This study investigated the proteomic profile of patients at risk for heart failure treated with spironolactone, with a special focus on DM (Fig. 3). The main findings of the study are: (i) patients with diabetes have a distinct proteome profile, whereby twenty-one biomarkers are higher, while five were lower compared to those without diabetes; (ii) cluster analysis revealed that the distinct proteomic profile is indicative of increased activation of inflammatory and proteolytic pathways in patients with diabetes; and (iii) diabetic status did not influence the treatment response to spironolactone with respect to change in the proteomic profile, as well as to the anti-fibrotic, anti-remodelling, blood pressure and natriuretic peptide lowering effects. It is unlikely that diabetic status modified the effect of spironolactone observed in the HOMAGE trial.

### Proteomic profile of patients with diabetes

Patients with DM had an up-regulation of inflammatory and proteolytic pathways, suggesting that inflammation and proteolysis mechanisms (i.e. an increased protein turn-over via the lysosomal proteolytic system) are active and distinctive for patients with DM compared to non-diabetic patients at risk of developing HF. Based on our data we can not state any causal relationship between these proteins and pathways and diabetes. However, it seems that the observations are independent of coronary artery disease, hypertension, BMI, age, sex and kidney function. Our findings are in line with a previous network and pathway analysis in patients with both diabetes and HF, showing a similar association between DM and inflammation, protein phosphorylation, and neutrophil degranulation mechanistic pathways [4]. The top 5 proteins we identified (GDF15, GAL4, IL1RT1, CTSD and SELE) are robustly associated with diabetes in a variety of studies, consistent with our findings [2–4].

*Growth differentiation factor 15 (GDF15)* is a protein which is upregulated as consequence of organ injury, as seen in heart and kidney disease [10]. In patients with HF, GDF15 is a strong prognostic marker [11]. However, in diabetic patients without HF, GDF15 may be associated with the effect of metformin. Metformin partly works through stimulation of GDF15 expression, which is associated with weight loss and improved insulin sensitivity in patients with DM [12, 13]. On the other hand, GDF15 levels are increased in patients with HF, and GDF15 is associated with diastolic dysfunction, as defined by an elevated E/e′ [14, 15], which was also observed in the patients with DM in our study.

*Galectin-4 (GAL4)* is a small lectin protein and is involved in protein trafficking from the inside of the cell towards the cell membrane. This protein was detected in all previous proteomics screenings in diabetic patients, confirming a strong association between DM and GAL4. One of the proteins which is trafficked by GAL4 is the protease dipeptidyl peptidase-4 (DPP4). DPP4 subsequently deactivates proglucagon-derived peptide glucagon-like peptide-1 (GLP1). Inhibitors of DPP4 and GLP1-analogues are both used for lowering glucose in patients with DM but do not improve HF outcomes. An increase in GAL4 leads to increased activity of DPP4, which reduces the activity of GLP1, contributing to insulin resistance and DM development [3]. Although the association between GAL4 and DM is established, the exact mechanistic and causal role remains unknown.

*Interleukin-1 receptor type 1 (IL1RT1)* is the receptor for IL1A, IL1B and IL1RA and is responsible for the IL1-dependent activation of the NFĸB pathway. However, recently IL1R1 has been identified as one of the key mediators in leptin sensitivity and IL1R1 deficiency leads to a higher degree of obesity and metabolic disturbance [16]. Pro-inflammatory signalling can have beneficial metabolic effects: for example, inflammatory signalling is essential for proper adipose tissue remodelling and expansion which prevents ectopic lipid accumulation and subsequent glucose intolerance [17, 18]. Cytokine resistance as a consequence of chronic systemic inflammation could be one of the mechanisms underlying the development of DM type 2. It may be that the higher levels of inflammatory proteins we observed could be partially explained by cytokine resistance in diabetic patients.

*Cathepsin D (CTSD)* is a lysosomal endopeptidase which is involved in intracellular protein turnover and extracellular matrix breakdown. Levels of CTSD have been associated with DM in patients with and without HF [2, 3, 19], and are positively associated with insulin resistance [20]. The family of cathepsins were well-represented in the network analysis, showing a link between inflammation and proteolysis. It has been suggested that CTSD acts as a mediator between obesity and systemic inflammation, which could contribute to the cytokine resistance mentioned above [21].

*E-selectin (SELE)* is a selectin cell adhesion molecule expressed only on endothelial cells activated by cytokines. Levels of SELE and other cellular adhesion molecules have previously been associated with DM, increasing HbA1c levels, blood pressure and microalbuminuria, all reflecting (early) endothelial dysfunction [22–24].

### Granulin as important node in the diabetes pathophysiology

Granulin was identified as a key hub in the network based on the proteomic profile of patients with DM. Granulin is cleaved from the precursor progranulin and their exact function remains unknown, but both forms have been implicated in inflammation and protein homeostasis via the lysosomal pathway (e.g. CTSD). Mechanistically (pro)granulin seems to play an important role in the proteomic network of diabetic patients and is related to insulin resistance, obesity, inflammation and proteolysis [25]. Mice deficient in *Grn* have lower body weight, fat mass and adipocyte size than wild-type mice receiving a standard diet. The loss of granulin protected the mice against obesity and insulin resistance induced by a high fat diet [25]. Granulin increases IL6-mediated expression of cytokine signalling 3 (SOCS3), which inhibits phosphorylation of insulin receptor substrate-1 (IRS1) [25]. A previous study has also inferred that granulin is a key hub in the proteomic profile of patients with HF and DM based on other biomarkers (although granulin itself was not actually measured) [4]. We now identify granulin as a biomarker which is elevated in diabetic patients.

### Spironolactone treatment in patients with diabetes

The study end of the HOMAGE trial was determined at 9 months, as it is expected that the biochemical response of spironolactone can be observed after 9 months of treatment. At the last visit of a patient, additional blood sampling was performed for proteomic analysis. Spironolactone did not significantly change the proteomic profiles of patients with diabetes, but yielded the same clinical benefits as in the complete HOMAGE cohort. The four proteins which were significantly modified in diabetic patients after 9 months of spironolactone treatment (COL1A1, PICP, SELE and MMP7) are altered in the same direction as in the complete population, indicating that the effects are not specific for diabetic patients [9]. These proteins are implicated in extracellular matrix metabolism (COL1A1, PICP), cell-cell adhesion (SELE) and proteolysis (MMP7). Furthermore, diabetes did not modify the effect of spironolactone on serum markers of collagen metabolism, indicating that patients with diabetes benefited equally from the antifibrotic effect of spironolactone compared to non-diabetics. No metabolic pathways detrimental for diabetic patients was worsened with spironolactone.

### Limitations

We tested the effect of spironolactone on multiple proteins applying a correction for test multiplicity to limit the occurrence of false positive findings; however, as HOMAGE was a randomized controlled trial, other proteins, whose levels were also significantly changed with spironolactone, might also be implicated in relevant pathways and biological processes and might be worth exploring in further studies. Additionally, many of the highlighted mechanisms should be furtherly replicated and confirmed at a cellular level. Our results and previous findings highlight granulin as an important hub in the pathophysiology of diabetes which should receive priority for further study. Due to the inclusion criteria of the HOMAGE study, the non-diabetic patient group mainly included patients with CAD, and thus we corrected all analyses for the presence of CAD in the patient groups. The HOMAGE study aimed to identify patients at risk for HF, therefore all of the included patients were above the age of 60 years old and had a cardiovascular risk profile. Therefore, these results can not be generalized to other populations. The Olink multiplex panels provide normalized protein expression (NPX) values, which are relative quantifications of the measured biomarkers. Absolute numbers of expression levels were not available.

## Conclusions

Diabetic patients at risk of developing HF have a proteomic bioprofile which differs from controls without diabetes, pointing towards mechanisms related to inflammation and protein homeostasis. Treatment with spironolactone did not influence the overall proteomic profile of diabetic compared to non-diabetic patients. Patients benefited similarly from spironolactone with respect to its anti-fibrotic, anti-remodeling, blood pressure and natriuretic peptide lowering effects regardless of the diabetes status.

## Supplementary Information

**Additional file 1: Table S1.** Protein names and respective Olink® panel sorted in alphabetical order. **Table S2. ** Pathway enrichment analysis using GO terms. **Table S3. ** Proteins which are significantly altered in diabetic patients after 9 months of spironolactone treatment. **Table S4. ** Interaction analysis between diabetic status of the patients and spironolactone treatment for the primary and secondary outcomes of the HOMAGE trial.

## Data Availability

The datasets used and/or analysed during the current study are available from the corresponding author on reasonable request.
