# Supplementary material for: Proteomic mechanistic profile of patients with diabetes at risk of developing heart failure: insights from the HOMAGE trial
Source: Cardiovasc Diabetol. 2021 Aug 9;20:163. doi: 10.1186/s12933-021-01357-9 (PMC8351439; doi:10.1186/s12933-021-01357-9)
Supplement: Supplementary file 1 — Additional file 1: Table S1. Protein names and respective Olink® panel sorted in alphabetical order. Table S2. Pathway enrichment analysis using GO terms. Table S3. Proteins which are significantly altered in diabetic patients after 9 months of spironolactone treatment. Table S4. Interaction analysis between diabetic status of the patients and spironolactone treatment for the primary and secondary outcomes of the HOMAGE trial. [file 12933_2021_1357_MOESM1_ESM.docx]

**Table S1.** *Protein names and respective Olink® panel sorted in alphabetical order*

| **Protein full name** | **Entry name** | **Olink® Panel*** | **Uniprot ID**** |
| --- | --- | --- | --- |
| Angiotensin-converting enzyme 2 | ACE2 | CVD II | Q9BYF1 |
| Adenosine Deaminase | ADA | INF | P00813 |
| Adisintegrin and metalloproteinase with thrombospondin motifs 13 | ADAMTS13 | CVD II | Q76LX8 |
| ADM | ADM | CVD II | P35318 |
| Agouti-related protein | AGRP | CVD II | O00253 |
| CD166 antigen | ALCAM | CVD III | Q13740 |
| Protein AMBP | AMBP | CVD II | P02760 |
| Angiopoietin-1 | ANG1 | CVD II | Q15389 |
| Aminopeptidase N | APN | CVD III | P15144 |
| Axin-1 | AXIN1 | INF | O15169 |
| Tyrosine-protein kinase receptor UFO | AXL | CVD III | P30530 |
| Azurocidin | AZU1 | CVD III | P20160 |
| Brain-derived neurotrophic factor | BDNF | INF | P23560 |
| Beta-nerve growth factor | BETANGF | INF | P01138 |
| Bleomycin hydrolase | BLMHYDROLASE | CVD III | Q13867 |
| Bone morphogenetic protein 6 | BMP6 | CVD II | P22004 |
| Natriuretic peptides B | BNP | CVD II | P16860 |
| Eukaryotic translation initiation factor 4E-binding protein 1 | BP1_4E | INF | Q13541 |
| Carbonic anhydrase 5A, mitochondrial | CA5A | CVD II | P35218 |
| Caspase-3 | CASP3 | CVD III | P42574 |
| Caspase 8 | CASP8 | INF | Q14790 |
| Eotaxin-1 | CCL11 | INF | P51671 |
| C-C motif chemokine 15 | CCL15 | CVD III | Q16663 |
| C-C motif chemokine 16 | CCL16 | CVD III | O15467 |
| C-C motif chemokine 17 | CCL17 | CVD II | Q92583 |
| C-C motif chemokine 19 | CCL19 | INF | Q99731 |
| C-C motif chemokine 20 | CCL20 | INF | P78556 |
| C-C motif chemikine 22 | CCL22 | CVD III | O00626 |
| C-C motif chemokine 23 | CCL23 | INF | P55773 |
| C-C motif chemokine 24 | CCL24 | CVD III | O00175 |
| C-C motif chemokine 25 | CCL25 | INF | O15444 |
| C-C motif chemokine 28 | CCL28 | INF | Q9NRJ3 |
| C-C motif chemokine 3 | CCL3 | CVD II | P10147 |
| C-C motif chemokine 4 | CCL4 | INF | P13236 |
| Scavenger receptor cysteine-rich type 1 protein M130 | CD163 | CVD III | Q86VB7 |
| Natural killer cell receptor 2B4 | CD244 | INF | Q9BZW8 |
| T-cell surface glycoprotein CD4 | CD4 | CVD II | P01730 |
| CD40L receptor | CD40 | INF | P25942 |
| CD40 ligand | CD40L | CVD II | P29965 |
| T-cell surface glycoprotein CD5 | CD5 | INF | P06127 |
| T cell surface glycoprotein CD6 | CD6 | INF | P30203 |
| SLAM family member 5 | CD84 | CVD II | Q9UIB8 |
| Complement component C1q receptor | CD93 | CVD III | Q9NPY3 |
| CUB domain-containing protein 1 | CDCP1 | INF | Q9H5V8 |
| Cadherin-5 | CDH5 | CVD III | P33151 |
| Carcinoembryonic antigenrelated cell adhesion molecule 8 | CEACAM8 | CVD II | P31997 |
| Chitinase-3-like protein 1 | CHI3L1 | CVD III | P36222 |
| Chitotriosidase-1 | CHIT1 | CVD III | Q13231 |
| Contactin-1 | CNTN1 | CVD III | Q12860 |
| Collagen alpha-1(I) chain | COL1A1 | CVD III | P02452 |
| Carboxypeptidase A1 | CPA1 | CVD III | P15085 |
| Carboxypeptidase B | CPB1 | CVD III | P15086 |
| Macrophage colony-stimulating factor 1 | CSF1 | INF | P09603 |
| Cystatin D | CST5 | INF | P28325 |
| Cystatin-B | CSTB | CVD III | P04080 |
| Chymotrypsin C | CTRC | CVD II | Q99895 |
| Cathepsin D | CTSD | CVD III | P07339 |
| Cathepsin L1 | CTSL1 | CVD II | P07711 |
| Cathepsin Z | CTSZ | CVD III | Q9UBR2 |
| Fractalkine | CX3CL1 | INF | P78423 |
| C-X-C motif chemokine 1 (CVD2) | CXCL1 | CVD II | P09341 |
| C-X-C motif chemokine 10 | CXCL10 | INF | P02778 |
| C-X-C motif chemokine 11 | CXCL11 | INF | O14625 |
| C-X-C motif chemokine 16 | CXCL16 | CVD III | Q9H2A7 |
| C-X-C motif chemokine 5 | CXCL5 | INF | P42830 |
| C-X-C motif chemokine 6 | CXCL6 | INF | P80162 |
| C-X-C motif chemokine 9 | CXCL9 | INF | Q07325 |
| Decorin | DCN | CVD II | P07585 |
| 2,4-dienoyl-CoA reductase, mitochondrial | DECR1 | CVD II | Q16698 |
| Dickkopf-related protein 1 | DKK1 | CVD II | O94907 |
| Azurocidin | DLK1 | CVD III | P80370 |
| Delta and Notch-like epidermal growth factor-related receptor | DNER | INF | Q8NFT8 |
| Epidermal growth factor receptor | EGFR | CVD III | P00533 |
| Protein S100-A12 | ENRAGE | INF | P80511 |
| Epithelial cell adhesion molecule | EPCAM | CVD III | P16422 |
| Ephrin type-B receptor 4 | EPHB4 | CVD III | P54760 |
| Fatty acid-binding protein, intestinal | FABP2 | CVD II | P12104 |
| Fatty acid-binding protein, adipocyte | FABP4 | CVD III | P15090 |
| Tumor necrosis factor receptor superfamily member 6 | FAS | CVD III | P25445 |
| Fibroblast growth factor 19 | FGF19 | INF | O95750 |
| Fibroblast growth factor 21 (CVD2) | FGF21 | CVD II | Q9NSA1 |
| Fibroblast growth factor 23 (CVD2) | FGF23 | CVD II | Q9GZV9 |
| Fibroblast growth factor 5 | FGF5 | INF | P12034 |
| Fms-related tyrosine kinase 3 ligand | FLT3L | INF | P49771 |
| Follistatin | FS | CVD II | P19883 |
| Galectin-3 | GAL3 | CVD III | P17931 |
| Galectin-4 | GAL4 | CVD III | P56470 |
| Galectin-9 | GAL9 | CVD II | O00182 |
| Growth/differentiation factor 15 | GDF15 | CVD III | Q99988 |
| Growth/differentiation factor 2 | GDF2 | CVD II | Q9UK05 |
| Growth hormone | GH | CVD II | P01241 |
| Gastric intrinsic factor | GIF | CVD II | P27352 |
| Lactoylglutathione lyase | GLO1 | CVD II | Q04760 |
| Granulins | GRN | CVD III | P28799 |
| Gastrotropin | GT | CVD II | P51161 |
| Hydroxyacid oxidase 1 | HAOX1 | CVD II | Q9UJM8 |
| Proheparin-binding EGF-like growth factor | HBEGF | CVD II | Q99075 |
| Glial cell line-derived neurotrophic factor | HGDNF | INF | P39905 |
| Hepatocyte growth factor | HGF | INF | P14210 |
| Heme oxygenase 1 | HO1 | CVD II | P09601 |
| Osteoclast-associated immunoglobulin-like receptor | HOSCAR | CVD II | Q8IYS5 |
| Heat shock 27 kDa protein | HSP27 | CVD II | P04792 |
| Intercellular adhesion molecule 2 | ICAM2 | CVD III | P13598 |
| Alpha-L-iduronidase | IDUA | CVD II | P35475 |
| Insulin-like growth factor-binding protein 1 | IGFBP1 | CVD III | P08833 |
| Insulin-like Growth Factor-Binding Protein 2 | IGFBP2 | CVD III | P18065 |
| Insulin-like growth factor-binding protein 7 | IGFBP7 | CVD III | Q16270 |
| Low affinity immunoglobulin gamma Fc region receptor II-b | IGGFCRECEPTORIIB | CVD II | P31994 |
| Interleukin-10 | IL10 | INF | P22301 |
| Interleukin-10 receptor subunit alpha | IL10RA | INF | Q13651 |
| Interleukin-10 receptor subunit beta | IL10RB | INF | Q08334 |
| Interleukin-12 subunit beta | IL12B | INF | P29460 |
| Interleukin-13 | IL13 | INF | P35225 |
| Interleukin-15 receptor subunit alpha | IL15RA | INF | Q13261 |
| Pro-interleukin-16 | IL16 | CVD II | Q14005 |
| Interleukin-17A | IL17A | INF | Q16552 |
| Interleukin-17C | IL17C | INF | Q9P0M4 |
| Interleukin-17D | IL17D | CVD II | Q8TAD2 |
| Interleukin-17 receptor A | IL17RA | CVD III | Q96F46 |
| Interleukin-18 (CVD2) | IL18 | CVD II | Q14116 |
| Interleukin-18-binding protein | IL18BP | CVD III | O95998 |
| Interleukin-18 receptor 1 | IL18R1 | INF | Q13478 |
| Interleukin-1 receptor antagonist protein | IL1RA | CVD II | P18510 |
| Interleukin-1 receptor-like 2 | IL1RL2 | CVD II | Q9HB29 |
| Interleukin-1 receptor type 1 | IL1RT1 | CVD III | P14778 |
| Interleukin-1 receptor type 2 | IL1RT2 | CVD III | P27930 |
| Interleukin-20 receptor subunit alpha | IL20RA | INF | Q9UHF4 |
| Interleukin-27 | IL27 | CVD II | Q8NEV9 |
| Interleukin-2 receptor subunit alpha | IL2RA | CVD III | P01589 |
| Interleukin-4 receptor subunit alpha | IL4RA | CVD II | P24394 |
| Interleukin-6 (CVD2) | IL6 | CVD II | P05231 |
| Interleukin-6 receptor subunit alpha | IL6RA | CVD III | P08887 |
| Interleukin-7 | IL7 | INF | P13232 |
| Interleukin-8 | IL8 | INF | P10145 |
| Melusin | ITGB1BP2 | CVD II | Q9UKP3 |
| Integrin beta-2 | ITGB2 | CVD III | P05107 |
| Junctional adhesion molecule A | JAMA | CVD III | Q9Y624 |
| Kidney injury molecule 1 | KIM1 | CVD II | Q96D42 |
| Kallikrein-6 | KLK6 | CVD III | Q92876 |
| Latency-associated peptide transforming growth factor beta 1 | LAPTGFBETA1 | INF | P01137 |
| Low-density lipoprotein receptor | LDLRECEPTOR | CVD III | P01130 |
| Leptin | LEP | CVD II | P41159 |
| Leukemia inhibitory factor receptor | LIFR | INF | P42702 |
| Lectin-like oxidized LDL receptor 1 | LOX1 | CVD II | P78380 |
| Lipoprotein lipase | LPL | CVD II | P06858 |
| Lymphotoxin-beta receptor | LTBR | CVD III | P36941 |
| Macrophage receptor MARCO | MARCO | CVD II | Q9UEW3 |
| Myoglobin | MB | CVD III | P02144 |
| Monocyte chemotactic protein 1 | MCP1_CVD3 | CVD III | P13500 |
| Monocyte chemotactic protein 2 | MCP2 | INF | P80075 |
| Monocyte chemotactic protein 3 | MCP3 | INF | P80098 |
| Monocyte chemotactic protein 4 | MCP4 | INF | Q99616 |
| Matrix extracellular phosphoglycoprotein | MEPE | CVD III | Q9NQ76 |
| Tyrosine-protein kinase Mer | MERTK | CVD II | Q12866 |
| Matrix metalloproteinase-1 | MMP1 | INF | P03956 |
| Matrix metalloproteinase-10 | MMP10 | INF | P09238 |
| Matrix metalloproteinase-12 | MMP12 | CVD II | P39900 |
| Matrix metalloproteinase-2 | MMP2 | CVD III | P08253 |
| Matrix metalloproteinase-3 | MMP3 | CVD III | P08254 |
| Matrix metalloproteinase-7 | MMP7 | CVD II | P09237 |
| Matrix metalloproteinase-9 | MMP9 | CVD III | P14780 |
| Myeloperoxidase | MPO | CVD III | P05164 |
| NF-kappa-B essential modulator | NEMO | CVD II | Q9Y6K9 |
| Neurogenic locus notch homolog protein 3 | NOTCH3 | CVD III | Q9UM47 |
| Neurotrophin-3 | NT3 | INF | P20783 |
| N-terminal prohormone brain natriuretic peptide | NTPROBNP | CVDII | P16860 |
| Osteoprotegerin | OPG_CVD3 | CVD III | O00300 |
| Osteopontin | OPN | CVD III | P10451 |
| Oncostatin-M | OSM | INF | P13725 |
| Plasminogen activator inhibitor 1 | PAI | CVD III | P05121 |
| Pappalysin-1 | PAPPA | CVD II | Q13219 |
| Proteinase-activated receptor 1 | PAR1 | CVD II | P25116 |
| Poly [ADP-ribose] polymerase 1 | PARP1 | CVD II | P09874 |
| Proprotein convertase subtilisin/kexin type 9 | PCSK9 | CVD III | Q8NBP7 |
| Platelet-derived growth factor subunit A | PDGFSUBUNITA | CVD III | P04085 |
| Platelet-derived growth factor subunit B | PDGFSUBUNITB | CVD II | P01127 |
| Programmed cell death 1 ligand 1 | PDL1 | INF | Q9NZQ7 |
| Programmed cell death 1 ligand 2 | PDL2 | CVD II | Q9BQ51 |
| Platelet endothelial cell adhesion molecule | PECAM1 | CVD III | P16284 |
| Peptidoglycan recognition protein 1 | PGLYRP1 | CVD III | O75594 |
| Elafin | PI3 | CVD III | P19957 |
| Polymeric immunoglobulin receptor | PIGR | CVD II | P01833 |
| Perlecan | PLC | CVD III | P98160 |
| Placenta growth factor | PLGF | CVD II | P49763 |
| Paraoxonase (PON 3) | PON3 | CVD III | Q15166 |
| Prolargin | PRELP | CVD II | P51888 |
| Brother of CDO | PROTEINBOC | CVD II | Q9BWV1 |
| Serine protease 27 | PRSS27 | CVD II | Q9BQR3 |
| Prostasin | PRSS8 | CVD II | Q16651 |
| Myeloblastin | PRTN3 | CVD III | P24158 |
| P-selectin glycoprotein ligand 1 | PSGL1 | CVD II | Q14242 |
| Pulmonary surfactant-associated protein D | PSPD | CVD III | P35247 |
| Pentraxin-related protein PTX3 | PTX3 | CVD II | P26022 |
| Receptor for advanced glycosylation end products | RAGE | CVD II | Q15109 |
| Retinoic acid receptor responder protein 2 | RARRES2 | CVD III | Q99969 |
| Renin | REN | CVD II | P00797 |
| Resistin | RETN | CVD III | Q9HD89 |
| Stem cell factor (CVD2) | SCF | CVD II | P21583 |
| Secretoglobin family 3A member 2 | SCGB3A2 | CVD III | Q96PL1 |
| E-selectin | SELE | CVD III | P16581 |
| P-selectin | SELP | CVD III | P16109 |
| Serpin A12 | SERPINA12 | CVD II | Q8IW75 |
| Tyrosine-protein phosphatase non-receptor type substrate 1 | SHPS1 | CVD III | P78324 |
| SIR2-like protein 2 | SIRT2 | INF | Q8IXJ6 |
| Signaling lymphocytic activation molecule | SLAMF1 | INF | Q13291 |
| SLAM family member 7 | SLAMF7 | CVD II | Q9NQ25 |
| Superoxide dismutase [Mn], mitochondrial | SOD2 | CVD II | P04179 |
| Sortilin | SORT1 | CVD II | Q99523 |
| Spondin-1 | SPON1 | CVD III | Q9HCB6 |
| Spondin-2 | SPON2 | CVD II | Q9BUD6 |
| Proto-oncogene tyrosine-protein kinase Src | SRC | CVD II | P12931 |
| Sulfotransferase 1A1 | ST1A1 | INF | P50225 |
| ST2 protein | ST2 | CVD III | Q01638 |
| STAM-binding protein | STAMPB | INF | O95630 |
| Serine/threonine-protein kinase 4 | STK4 | CVD II | Q13043 |
| Tissue factor | TF | CVD II | P13726 |
| Trefoil factor 3 | TFF3 | CVD III | Q07654 |
| Tissue factor pathway inhibitor | TFPI | CVD III | P10646 |
| Transforming growth factor alpha | TGFALPHA | INF | P01135 |
| Protein-glutamine gamma-glutamyltransferase 2 | TGM2 | CVD II | P21980 |
| Thrombospondin-2 | THBS2 | CVD II | P35442 |
| Thrombopoietin | THPO | CVD II | P40225 |
| Angiopoietin-1 receptor | TIE2 | CVD II | Q02763 |
| Metalloproteinase inhibitor 4 | TIMP4 | CVD III | Q99727 |
| Trem-like transcript 2 protein | TLT2 | CVD III | Q5T2D2 |
| Thrombomodulin | TM | CVD II | P07204 |
| Tumor necrosis factor | TNF | INF | P01375 |
| TNF-beta | TNFB | INF | P01374 |
| Tumor necrosis factor receptor 1 | TNFR1 | CVD III | P19438 |
| Tumor necrosis factor receptor 2 | TNFR2 | CVD III | P20333 |
| Tumor necrosis factor receptor superfamily member 10A | TNFRSF10A | CVD II | O00220 |
| Tumor necrosis factor receptor superfamily member 10C | TNFRSF10C | CVD III | O14798 |
| Tumor necrosis factor receptor superfamily member 11A | TNFRSF11A | CVD II | Q9Y6Q6 |
| Tumor necrosis factor receptor superfamily member 13B | TNFRSF13B | CVD II | O14836 |
| Tumor necrosis factor receptor superfamily member 14 | TNFRSF14 | CVD III | Q92956 |
| Tumor necrosis factor receptor superfamily member 9 | TNFRSF9 | INF | Q07011 |
| Tumor necrosis factor ligand superfamily member 13B | TNFSF13B | CVD III | Q9Y275 |
| Tumor necrosis factor ligand superfamily member 14 | TNFSF14 | INF | O43557 |
| Tissue-type plasminogen activator | TPA | CVD III | P00750 |
| Transferrin receptor protein 1 | TR | CVD III | P02786 |
| TNF-related apoptosis-inducing ligand | TRAIL | INF | P50591 |
| TNF-related apoptosis-inducing ligand receptor 2 | TRAILR2 | CVD II | O14763 |
| TNF-related activation-induced cytokine | TRANCE | INF | O14788 |
| Tartrate-resistant acid phosphatase type 5 | TRAP | CVD III | P13686 |
| Tumor necrosis factor (Ligand) superfamily, member 12 | TWEAK | INF | O43508 |
| Urokinase-type plasminogen activator | UPA_CVD3 | CVD III | P00749 |
| Urokinase plasminogen activator surface receptor | UPAR | CVD III | Q03405 |
| Vascular endothelial growth factor A | VEGFA | INF | P15692 |
| Vascular endothelial growth factor D | VEGFD | CVD II | O43915 |
| V-set and immunoglobulin domain-containing protein 2 | VSIG2 | CVD II | Q96IQ7 |
| von Willebrand factor | VWF | CVD III | P04275 |
| Lymphotactin | XCL1 | CVD II | P47992 |

**Table S2.** *Pathway enrichment analysis using GO terms.*

| **GO-term** | **Pathway** | **FDRq** | **Included proteins** |
| --- | --- | --- | --- |
| GO:0034097 | Response to cytokine | 0.0000269 | *CCL15, CCL11, CXCL10, SELE, PSGL1, IL1R1, IL10, LIFR, IL18R1, OPG, COL1A1* |
| GO:0071345 | Cellular response to cytokine stimulus | 0.0000472 | *CCL15, CCL11, CXCL10, PSGL1, IL1R1, IL10, LIFR, IL18R1, OPG, COL1A1* |
| GO:0050900 | Leukocyte migration | 0.0000472 | *CCL15, CCL11, CXCL10, SELE, PSGL1, IL10, COL1A1* |
| GO:0006954 | Inflammatory response | 0.0000472 | *CCL15, CCL11, CXCL10, SELE, IL1R1, IL10, IL18R1, OPG* |
| GO:0002376 | Immune system process | 0.0000472 | *CCL15, CCL11, CXCL10, SELE, PSGL1, IL1R1, IL10, IL18R1, FST, OPG, COL1A1, CTSD, CTSZ, GRN* |
| GO:0019221 | Cytokine-mediated signalling pathway | 0.00023 | *CCL15, CCL11, CXCL10, IL1R1, IL10, LIFR, IL18R1, OPG* |
| GO:0010469 | Regulation of signaling receptor activity | 0.0011 | *CCL15, CCL11, CXCL10, IL10, GRN, GDF15, OPG* |
| GO:0034612 | Response to tumor necrosis factor | 0.0012 | *CCL15, CCL11, SELE, OPG, COL1A1* |
| GO:0006955 | Immune response | 0.0015 | *CCL15, CCL11, CXCL10, IL1R1, IL10, IL18R1, OPG, CTSD, CTSZ, GRN* |
| GO:0030595 | Leukocyte chemotaxis | 0.0027 | *CCL15, CCL11, CXCL10, IL10* |

**Table S3.** *Proteins which are significantly altered in diabetic patients after 9 months of spironolactone treatment.*

|  | **Co-efficient (95% CI)** | **p-value** |
| --- | --- | --- |
| *Decreased after 9 months of spironolactone* | | |
| **COL1A1** | -0.14 (-0.2 – -0.08) | 0.00001 |
| **PICP** | -8.03 (-11.85 – -4.22) | 0.00004 |
| **SELE** | -0.09 (-0.16 – -0.02) | 0.01 |
| *Increased after 9 months of spironolactone* | | |
| **MMP7** | 0.1 (0.03 – 0.18) | 0.009 |

**Table S4.** *Interaction analysis between diabetic status of the patients and spironolactone treatment for the primary and secondary outcomes of the HOMAGE trial.*

|  | **No diabetes**  **Co-efficient (95% CI)** | **Diabetes**  **Co-efficient (95% CI)** | **Interaction p-value (diabetes*spironolactone)** |
| --- | --- | --- | --- |
| **PICP** | -9.41 (-14.42--4.41) | -6.13 (-12.05--0.21) | 0.39 |
| **PIIINP** | -0.09 (-0.48-0.31) | -0.24 (-0.69-0.2) | 0.6 |
| **CITP** | 0.34 (-0.06-0.74) | -0.28 (-1.26-0.7) | 0.19 |
| **NT-proBNP** | -5.21 (-9.77--0.65) | -10.55 (-23.81-2.7) | 0.39 |
| **Systolic blood pressure** | -10.89 (-14.4--7.38) | -8.63 (-13--4.27) | 0.45 |
| **Diastolic blood pressure** | -3.79 (-5.72--1.85) | -1.92 (-4.38-0.52) | 0.29 |
| **Left atrial volume** | -2.9 (-4.42--1.38) | -1.36 (-3.47-0.76) | 0.23 |
| **Left ventricular mass** | -3.4 (-6.12--0.67) | -0.04 (-4.1-4.01) | 0.16 |
